# Dietary PUFAs attenuate NLRP3 inflammasome activation via enhancing macrophage autophagy

**DOI:** 10.1194/jlr.M075879

**Published:** 2017-07-20

**Authors:** Lulu Shen, Yan Yang, Tiantong Ou, Chia-Chi C. Key, Sarah H. Tong, Russel C. Sequeira, Jonathan M. Nelson, Yan Nie, Zhan Wang, Elena Boudyguina, Swapnil V. Shewale, Xuewei Zhu

**Affiliations:** Department of Internal Medicine,* Section on Molecular Medicine, Wake Forest School of Medicine, Winston-Salem, NC; Prestige Department of Poultry Science,† North Carolina State University, Raleigh, NC

**Keywords:** inflammation, atherosclerosis, omega-3 fatty acids, fish oil

## Abstract

Dietary PUFAs reduce atherosclerosis and macrophage inflammation, but how nucleotide-binding oligomerization domain leucine-rich repeat-containing receptor protein (NLRP3) inflammasome activation and autophagy influence PUFA-mediated atheroprotection is poorly understood. We fed Ldlr^−/−^ mice diets containing 10% (calories) palm oil (PO) and 0.2% cholesterol, supplemented with an additional 10% of calories as PO, fish oil (FO), echium oil (EO, containing 18:4 n-3), or borage oil (BO, containing 18:3 n-6). Inflammasome activation, autophagic flux, and mitochondrial function were measured in peritoneal macrophages, blood monocytes, or liver from diet-fed mice. Compared with PO, dietary PUFAs (FO, EO, or BO) markedly inhibited inflammasome activation, shown by *1*) less macrophage IL-1β secretion and caspase-1 cleavage in response to NLRP3 inflammasome activators, *2*) less IL-1β secretion and caspase-1 cleavage from liver or hepatocytes in response to lipopolysaccharide (LPS), and *3*) attenuated caspase-1 activity in blood monocytes. Furthermore, PUFA-enriched diets increased LC3-II expression in macrophage, aorta, and liver samples and reduced numbers of dysfunctional mitochondria in macrophages in response to LPS and palmitate, suggesting enhanced autophagic activation. Dietary PUFAs did not attenuate NLRP3 inflammasome activation in atg5-deficient macrophages, indicating that autophagic activation is critical for the PUFA-mediated inflammasome inactivation. In conclusion, dietary PUFAs reduce atherosclerosis, in part, by activation of macrophage autophagy and attenuation of NLRP3 inflammasome activation.

Cardiovascular disease, mainly caused by atherosclerosis, is a leading cause of mortality worldwide. Macrophage inflammation plays a critical role in all stages of atherosclerosis. Macrophages express pattern recognition receptors, including well-characterized Toll-like receptors (TLRs) and intracellular Nod-like receptors (NLRs). The NLR family contains proteins that form large multimeric protein complexes, termed inflammasomes (1–3). Inflammasomes can be activated by multiple types of tissue damage or by pathogen-associated molecular patterns, resulting in autocatalytic cleavage of pro-caspase-1 and processing/maturation and secretion of the proinflammatory cytokines interleukin-1β (IL-1β) and IL-18. Atherogenic factors such as cholesterol crystals (4, 5) can activate the nucleotide-binding oligomerization-domain leucine-rich repeat containing receptor protein (NLRP3) inflammasome in macrophages via disruption of lysosomal membranes. Oxidized LDL (oxLDL) also can activate the NLRP3 inflammasome by priming macrophages through TLRs and activating NLRP3 by forming cholesterol crystals in lysosomes in a CD36-dependent manner (6). Furthermore, saturated FAs such as palmitate can switch on the NLRP3 inflammasome via an AMP-activated protein kinase-autophagy pathway that activates macrophage NLRP3 inflammasomes (7). Lastly, mice with NLRP3 or IL-1β deficiency in bone marrow cells develop less inflammation and atherosclerosis under hypercholesterolemic conditions (4, 8), supporting the correlation between activation of the NLRP3 inflammasome in macrophages and development of atherosclerosis.

Autophagy is a highly conserved homeostatic process for degradation of cytosolic macromolecules, damaged organelles, and pathogens. Interestingly, progression of atherosclerosis leads to progressive deterioration of macrophage autophagy (9). This deficiency impairs cholesterol efflux (10), increases apoptosis and oxidative stress (11), enhances NLRP3 inflammasome activation (9), and promotes atherosclerotic plaque progression. Autophagy negatively regulates inflammasomes through multiple mechanisms, including *1*) targeting pro-IL-1β (12), *2*) ubiquitinating inflammasomes for degradation (13), and *3*) removing damaged mitochondria and suppressing release of mitochondrial reactive oxygen species (ROS) and DNA (14, 15).

Fish oil (FO) and n-3 PUFAs reduce atherosclerosis in several animal models, including monkeys and transgenic mice, compared with saturated fat diets (16–19). In addition to n-3 PUFAs, n-6 PUFAs are also atheroprotective in mouse models of atherosclerosis, in nonhuman primates, and in human populations (20, 21). Most PUFA-enriched fats are derived from botanical oils; these consist of 18-carbon PUFAs that are poorly converted to ≥20-carbon PUFAs by the rate-limiting enzyme delta-6 desaturase (D6D). We have demonstrated that botanical oils enriched in 18-carbon FAs beyond D6D [echium oil (EO), containing 18:4 n-3, and borage oil (BO), containing 18:3 n-6] result in efficient conversion and enrichment of their respective ≥20-carbon PUFAs, 20:5 n-3 and 20:4 n-6. Furthermore, compared with the saturated/monounsaturated FA-enriched palm oil (PO), dietary enrichment with EO or BO reduced plasma cholesterol concentrations, splenic monocytosis, neutrophilia, monocyte trafficking into aortic plaques, and atherosclerosis, similar to results observed with FO consumption (22–24).

In this study, we examined the effects of dietary PUFAs on macrophage autophagy and NLRP3 inflammasome activation. Our results suggest that dietary n-3 (FO and EO) and n-6 PUFAs (BO) markedly enhance activation of macrophage autophagy, improve mitochondrial function, and attenuate NLRP3 inflammasome activation, all of which partially explain their protective effects against atherosclerosis.

## MATERIALS AND METHODS

### Dietary oils

Seed oil of Borago officinalis L., a member of the Boraginaceae family, was generously donated by Nordic Naturals (Watsonville, CA). The seed oil of Echium plantagineum L., a member of the Boraginaceae family, was a generous gift from Croda Europe Ltd. (Leek, Staffordshire, UK). All oils were authenticated by the Wake Forest University Center for Botanical Lipids and Inflammatory Disease Prevention. The seed oil of the palm, Elaeis guineensis Jacq, a member of the Arecaceae family, was purchased from Shay and Co. (Portland, OR). A certificate of analysis is on file for reference, and retention samples of both seed oils are deposited, at the Wake Forest School of Medicine. The fish oil source was Brevoortia tyrannis Latrobe, a member of the Clupeidae family, and was manufactured and generously provided by Omega Protein (Houston, TX), with a report of analysis on file for reference.

### Animals and atherogenic diets

Ldlr^−/−^ (stock 002207), LysMcre (stock 004781), and C57BL/6 (stock 000664) mice were purchased from Jackson Laboratories. Atg5 flox/flox mice were purchased from Riken BioResource Center (stock RBRC02975). We crossed atg5 flox/flox mice and LysMcre mice to generate atg5 macrophage-specific KO (MSKO) mice. Atg5 flox/flox mice were used as WT controls. Mice were housed in a specific pathogen-free facility on a 12 h light/dark cycle. At 8 weeks of age, mice were randomly assigned to one of four groups consuming atherogenic diets containing 10% calories as PO and 0.2% cholesterol, supplemented with an additional 10% of calories as *1*) PO, *2*) BO (18:3 n-6 enriched), *3*) EO (18:4 n-3 enriched), or *4*) FO (20:5 n-3 and 22:6 n-3 enriched) for an additional 8–16 weeks. We also performed bone marrow transplantation as previously described (24). Briefly, bone marrow cells were harvested from cleaned femurs and tibias of male WT and atg5 MSKO mice and injected into irradiated (900 rads) Ldlr^−/−^ mice (stock 002207) (7 × 10^6^ bone marrow cells per mouse). After 7 weeks of recovery from bone marrow transplantation, mice were randomly assigned to one of the four atherogenic diets as described above for an additional 16 weeks. The experimental protocols were approved by the Wake Forest University Animal Care and Use Committee. Atherogenic diets were prepared by the diet kitchen in the Department of Pathology at Wake Forest School of Medicine as previously described (25). Detailed composition and quality control data for similar atherogenic diets have been published (23, 24, 26).

### FA analysis

Lipids of lyophilized diets, peritoneal macrophages, and liver were extracted using the Bligh-Dyer method (27). The lipid extracts were transmethylated using boron trifluoride, and the percentage of FA composition was quantified as described previously (25).

### Peritoneal macrophage culture

Elicited peritoneal macrophages were collected 3 days after injection of 1 ml of 10% thioglycolate into the peritoneal cavity, as described previously (28). Resident peritoneal macrophages were harvested from mice by directly flushing the peritoneal cavity with cold PBS. The peritoneal cells were plated in RPMI-1640 media containing 100 U/ml penicillin, 100 µg/ml streptomycin, and 1% Nutridoma SP media (Roche Applied Science, no. 11011375001). After a 2 h incubation, floating cells were removed by washing with PBS, and adherent macrophages were used for experiments (28).

### Primary hepatocyte isolation and LPS stimulation

Primary hepatocyte isolation from atherogenic diet-fed mice was performed with a slight modification from a previous study (29). The day before hepatocyte isolation, culture plates were coated with collagen (Sigma-Aldrich, no. C3867) at 10 μg/cm^2^ for 2 h at 37°C. The day of hepatocyte isolation, perfusion (10 mM HEPES, 0.5 mM EDTA) and digestion (5 mM CaCl_2_, 0.05% type I collagenase) solutions were freshly made in Ca_2_^+^ and Mg_2_^+^ free Hanks’ balanced salt solution (Life Technologies, no. 14185-052) and placed in a 45°C water bath. Mice were anesthetized with ketamine/xylazine, and a midline laparotomy was performed. The portal vein was visualized, and a 23 gauge needle was inserted. The inferior vena cava was cut, and the liver was perfused with perfusion solution at a flow rate of 4 ml per minute for 6 min, followed by perfusion with digestion solution at a flow rate of 4 ml per minute for 8 min. The gall bladder was removed, and the liver was transferred into a 100 mm dish containing 10 ml of warm digestion solution for 4 min to continue the digestion. The liver was gently minced with scissors. The cell suspension was filtered through a 200 µm nylon mesh (Fisher Scientific, no. NC0148096), and cells were washed twice in ice-cold William’s E medium (Life Technologies, no. 12551-032) at 50 rpm for 5 min. The primary hepatocytes were cultured in William’s E medium containing 100 U/ml of penicillin and 100 μg/ml of streptomycin at 0.3 × 10^6^ cells per well (6-well plates) for 2 h before being treated with or without 100 ng/ml lipopolysaccharide (LPS) for 3 or 24 h.

### Macrophage inflammatory gene expression analysis

Elicited peritoneal macrophages were primed with 200 ng/ml LPS for 2 h. Macrophages were then treated with or without 5 mM ATP (Sigma-Aldrich, no. A6419) for 1 h, 25 µg/ml ox-LDL for 24 h, or 200 μM palmitate-BSA for 24 h. In some experiments, after LPS priming, macrophages were treated with or without 5 mM ATP plus 3-methyladenine (3-MA; Sigma-Aldrich, no. M9281) for 1 h or 200 μM palmitate-BSA plus 3-MA for 24 h. The culture supernatants were collected and stored at −80°C for cytokine ELISA (BD Bioscience). Resident peritoneal macrophages from diet-fed Ldlr^−/−^ mice receiving bone marrow from WT and atg5 MSKO mice were stimulated with or without 100 ng/ml LPS for 3 h. Total RNA was isolated from macrophages using TRIzol reagent (Fisher Scientific, no. 15596026). cDNA preparation and real-time PCR were conducted as described previously (28). Total cell protein was harvested from macrophages using RIPA buffer containing protease inhibitor cocktails (Roche, no. 05892791001).

### Liver tissue culture

Liver tissues were isolated and then washed in cold PBS supplemented with penicillin and streptomycin. The liver tissues were chopped and cultured in 12-well plates in opti-MEM medium (Fisher Scientific, no. 31985070) supplemented with 100 U/ml penicillin and 100 µg/ml streptomycin. After 24 h, supernatants were collected and stored at −80°C until analyzed.

### Plasma concentration of cytokines after LPS injection into mice

At 8 weeks of age, C57BL/6 mice were randomly assigned to one of four atherogenic diet groups as described above. After 10 weeks of diet feeding, mice were injected in the peritoneal cavity with LPS (3 mg/kg body weight, Sigma-Aldrich, no. L6143) or saline (vehicle). One hour later, 100 μl of blood was drawn via the retro-orbital vein. Three hours after injection, mice were euthanized and bled by cardiac puncture. Plasma was isolated by centrifugation at 12,000 *g* for 15 min at 4°C and stored at −80°C until analyses were performed.

### Mitochondrial function and mitochondrial measurement of ROS

For measurement of mitochondrial mass, peritoneal cells were stained for 15 min at 37°C with 25 nM MitoTracker Green FM (Fisher Scientific, no. M7514) and MitoTracker Deep Red FM (Fisher Scientific, no. M22426). Mitochondrial ROS was measured in cells by MitoSOX (Fisher Scientific, no. M36008) staining (5 µM for 15 min at 37°C). Cells were washed with PBS and scraped off the dishes. Data were acquired with a FACSCanto II (BD Biosciences) and were analyzed with FlowJo analytical software (TreeStar).

### Macrophage assays for production of ROS

Peritoneal macrophages were incubated in 96-well plates and treated with 100 ng/ml LPS or LPS plus palmitate (200 μM) in the presence of 5- (and 6-) chloromethyl-2’,7’-dichlorodihrofluorescein diacetate, acetylester (DCFDA; 50 μM) (Sigma-Aldrich, no. D6883) for 3 h. Production of ROS in macrophages was determined by hydrolysis of DCFDA to fluorescent 2’,7’-dichlorofluorescein. DCFDA conversion was kinetically measured every 30 min in a microplate reader (BMG LABTECH) at 488 nm excitation and 535 nm emission.

### Blood cell flow cytometry

Peripheral blood was collected by submandibular vein puncture into heparin-containing tubes. Red blood cells were removed from flow cytometry preparations by treatment with ACK lysing buffer (Gibco, no. A10492). The remaining white blood cells were resuspended in Hanks’ balanced salt solution (0.1% BSA, 5 mM EDTA). Blood cells were then incubated with FLICA 660-YVAD-FMK (ImmunoChemistry Technologies, no. 9122) at 1:150 dilution in RPMI-1640 medium plus 5% FBS at 37°C for 1 h, followed by staining for monocytes and neutrophils using a cocktail of antibodies against CD45-APC (BD Pharmingen, no. 559864), Ly6-C/G-PerCP-Cy5.5 (BioLegend, no. 108427), and CD115-PE (eBioscience, no. 12-1152). Monocytes were identified as CD45^+^CD115^+^ and further separated into Ly6C^high^ and Ly6C^low^ subsets, and neutrophils were identified as CD45^+^CD115^−^Ly6G^+^. Data were acquired on a BD FACS Canto II instrument (BD Biosciences, Becton, Dickinson and Co., Franklin Lakes, NJ) and analyzed using FACSDiva software (version 6.1.3, BD Biosciences).

### ELISA

Supernatants from cell culture, plasma, or tissue cultures were assayed for mouse IL-1β (BD Biosciences, no. 559603), mouse IL-18 (eBioscience, no. BMS618/3) and IL-6 (BD Biosciences, no. 555240), according to the manufacturer’s instructions.

### Western blotting

Western blots were performed using specific antibodies against caspase-1p20 (Adipogen, no. AG-20B-0042), LC-3 (Novus Biologicals, no. NB100-2220), P62 (Novus Biologicals, no. NBP1-48320), NLRP3 (Adipogen, no. AG-20B-0014), β-actin (Sigma, no. A5441), and GAPDH (Santa Cruz, no. sc-32233). Blots were developed using HRP-linked secondary antibody. Immunoblots were visualized with the Supersignal substrate system (Pierce, no. 34078), and chemiluminescence was captured with an LSA-3000 imaging system (Fujifilm Life Science) or Kodak X-Omat XLS-1 film.

### Statistical analysis

Data are presented as the mean ± SEM unless indicated otherwise. Differences were compared with two-tailed Student’s *t* test or one-way ANOVA using GraphPad Prism software. *P* < 0.05 was considered statistically significant.

## RESULTS

### FA percentage compositions in diet-fed mouse macrophages and liver

Atherogenic diet FA compositions are given in **Table 1** and are similar to those published previously (23, 24). Dietary FA compositions showed relative enrichment of 18-carbon FAs beyond D6D in the BO (11.5% 18:3 n-6) and EO (6.5% 18:4 n-3) atherogenic diets. Similar to what we found in red blood cells (23, 24), 18:3 n-3 derived from EO was sufficiently elongated-desaturated to 20:5 n-3 (EPA) and 18:3 n-6 derived from BO was sufficiently elongated-desaturated to 20:3 n-6 (dihomo-γ-linolenic acid; DGLA) and 20:4 n-6 (arachidonic acid; AA) in thioglycollate-elicited peritoneal macrophages from 16-week diet-fed mice (**Table 2**). We also noticed that 22:5 n-3 (docosapentaenoic acid; DPA), elongated-desaturated from EPA, was the most abundant n-3 PUFA in both EO- and FO-fed mouse macrophages. We increased 22:6 n-3 (DHA) only in FO-fed mouse macrophages. In liver, 18-carbon FAs beyond D6D were sufficiently elongated and desaturated to longer-chain counterparts (**Table 3**). However, in general, PUFA enrichment in liver was low for all diet groups in relation to macrophages, which is likely due to the low enrichment of PUFAs in liver cholesteryl ester and triglyceride (23). Together, our results indicate that dietary enrichment of 18-carbon FAs beyond D6D is sufficient to result in membrane enrichment in their respective 20- or 22-carbon chain counterparts.

**TABLE 1.** FA composition and total energy equivalence of individual FAs in each atherogenic diet

	PO		FO		EO		BO	
FA	% FA	% EE	% FA	% EE	% FA	% EE	% FA	% EE
Palmitic acid (C16:0)	43.4	8.68	30.8	6.16	24.8	4.96	26.1	5.22
Palmitoleic acid (C16:1)	0.3	0.06	5	1	0.4	0.08	0.4	0.08
Stearic acid (C18:0)	4.4	0.88	4.3	0.86	3.9	0.78	4	0.8
Oleic acid (C18:1 n-9)	36.5	7.3	23.3	4.66	25.3	5.06	25.8	5.16
LA (C18:2 n-6)	11.2	2.24	5.7	1.14	14.1	2.82	25.5	5.1
ALA (C18:3 n-3)	0.3	0.06	0.9	0.18	16.5	3.3	0.4	0.08
GLA (C18:3 n-6)	0	0	0.2	0.04	5	1	11.5	2.3
SDA (C18:4 n-3)	0	0	1.7	0.34	6.5	1.3	0.1	0.02
Euric acid (C22:1 n-9)	0	0	0	0	0.3	0.06	1.2	0.24
EPA (C20:5 n-3)	0.3	0.06	7.3	1.46	0.3	0.06	0.3	0.06
DHA (C22:6 n-3)	0.3	0.06	7.6	1.52	0.4	0.08	0.4	0.08

Diets contained 0.2% cholesterol plus 10% calories as PO plus 10% calories as PO, BO, EO, or FO. Percentage of FA composition (% FA) of PO, BO, EO, and FO diets determined using GC/LC. Percentage of total energy equivalence (% EE) for individual FAs was calculated using total energy derived from FAs (i.e., 20%)/diet and percentage FA composition of respective diet. ALA, alpha-linoleic acid; EE, energy equivalence; GLA, gamma-linolenic acid; LA, linolenic acid; SDA, stearidonic acid.

**TABLE 2.** Peritoneal macrophage FA composition

	% FA			
FA	PO	FO	EO	BO
Palmitic acid (C16:0)	20.5	16.9	18.7	13.7
Oleic acid (C18:1 n-9)	25.4	17.5	15.3	14.2
Linoleic acid (C18:2 n-6)	6.5	5.4	6.3	6.9
ALA (C18:3 n-3)	0.1	0.2	0.4	0.2
GLA (C18:3 n-6)	0.4	0	0.1	0.1
DGLA (C20:3 n-6)	1.8	1.0	4.6	6.4
AA (C20:4 n-6)	9.0	4.8	9.1	13.7
EPA (C20:5 n-3)	0.2	5.3	1.1	0
DPA (C22:5 n-3)	1.8	13.7	6.3	1.2
DHA (C22:6 n-3)	4.4	10.3	3.4	3.0

**#x2212/#x2212:** Thioglycollate-elicited peritoneal macrophages were isolated from Ldlr^−/−^ mice fed the indicated atherogenic diets for 16 weeks. FA percentage distribution was measured using pooled cell lysates from three mice per diet group as described in the Materials and Methods section. Data for individual FAs are expressed as percentage composition of total FAs.

**TABLE 3.** Liver FA composition

	% FA			
FA	PO	FO	EO	BO
Palmitic acid (C16:0)	21.47 ± 0.26^a^	23.90 ± 0.34^a^	21.89 ± 0.64^a^	21.19 ± 1.14^a^
Oleic acid (C18:1 n-9)	48.39 ± 0.79^a^	35.26 ± 0.67^b^	39.98 ± 1.83^b,c^	42.02 ± 0.15^c^
Linoleic acid (C18:2 n-6)	4.12 ± 0.49^a^	4.15 ± 0.22^a^	6.93 ± 1.09^a,b^	10.66 ± 2.46^b^
ALA (C18:3 n-3)	0.05 ± 0.02^a^	0.33 ± 0.03^a^	3.60 ± 0.74^b^	0.05 ± 0.02^a^
GLA (C18:3 n-6)	0.09 ± 0.01^a^	0.05 ± 0.01^a^	0.56 ± 0.08^a^	1.51 ± 0.33^b^
DGLA (C20:3 n-6)	0.53 ± 0.04^a^	0.37 ± 0.02^a^	1.29 ± 0.11^b^	1.56 ± 0.27^b^
AA (C20:4 n-6)	2.08 ± 0.14^a,b,c^	1.54 ± 0.06^b^	2.51 ± 0.24^c^	4.31 ± 0.15^d^
EPA (C20:5 n-3)	0.16 ± 0.02^a^	5.80 ± 0.26^b^	2.02 ± 0.23^c^	0.10 ± 0.01^a^
DPA (C22:5 n-3)	0.09 ± 0.01^a^	1.43 ± 0.12^b^	0.71 ± 0.12^c^	0.11 ± 0.04^a^
DHA (C22:6 n-3)	1.65 ± 0.15^a^	8.01 ± 0.57^b^	2.91 ± 0.37^a^	1.67 ± 0.29^a^

**#x2212/#x2212:** Liver (n = 3 mice per diet group) were isolated from Ldlr^−/−^ mice fed the indicated atherogenic diets for 16 weeks. FA percentage distribution was measured as described in the Materials and Methods section. Data for individual FAs are expressed as percentage composition of total FAs. Values with different superscripts differ significantly (*P* < 0.05).

### Dietary supplementation of PUFAs reduced activation of the macrophage inflammasome

**Fig. 1.**
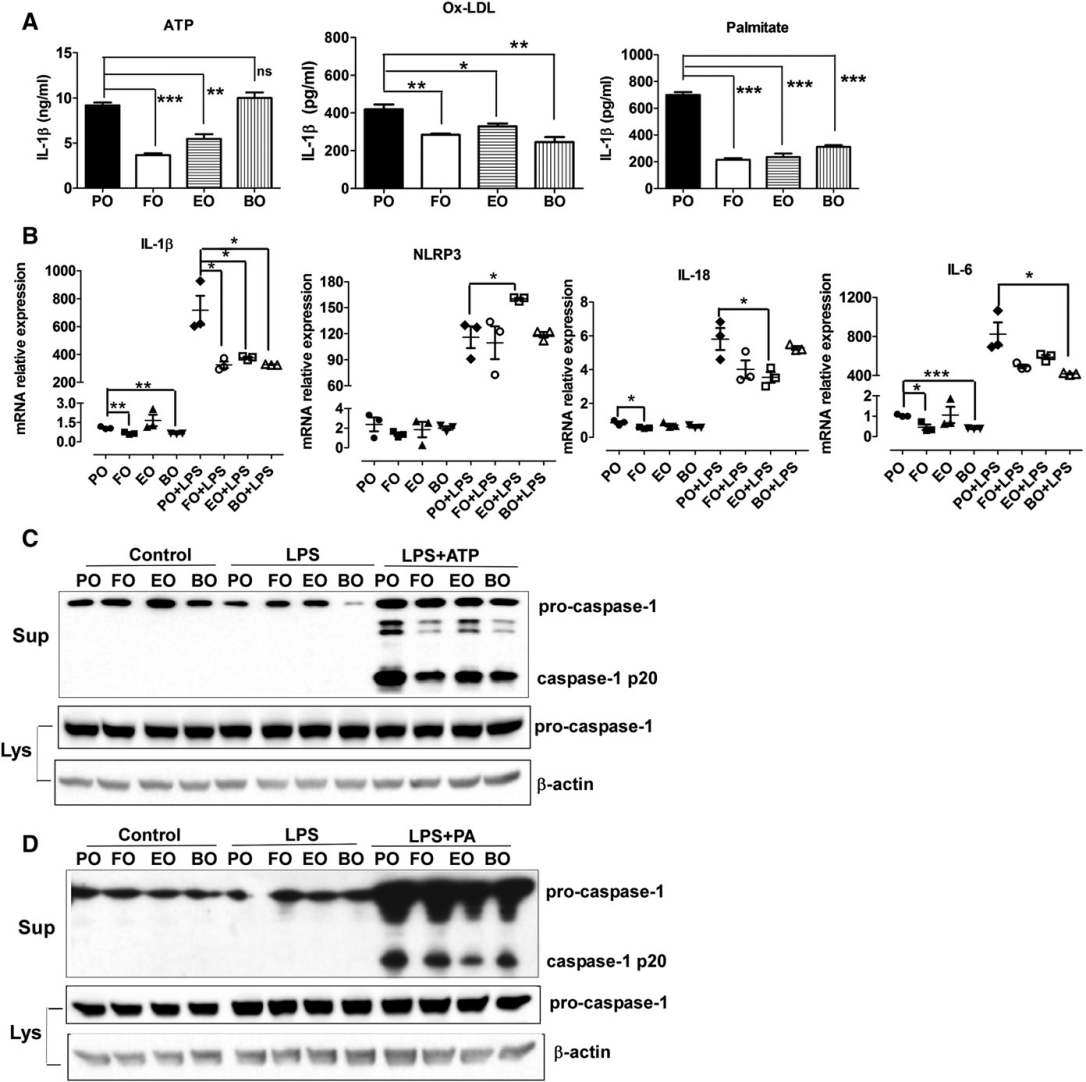
Dietary PUFAs reduced activation of the macrophage NLRP3 inflammasome. Female Ldlr^−/−^ mice (8 weeks old) were fed atherogenic diets for 10 weeks. Thioglycollated-elicited peritoneal macrophages were isolated, primed with 200 ng/ml LPS for 2 h, and treated with 5 mM ATP for 1 h, 25 µg/ml oxidized LDL (oxLDL) for 24 h, or 200 μM palmitate-BSA for 24 h. A: IL-1β secretion in culture media was assayed using ELISA. B: mRNA expression of NLRP3 inflammasome components and IL-6 was measured by real-time PCR. C: Caspase-1 cleavage in response to ATP simulation was analyzed by immunoblotting. D: Caspase-1 cleavage in response to palmitate stimulation was analyzed by immunoblotting. *P < 0.05; **P < 0.01; ***P < 0.001. Lys, cell lysates; ns, nonsignificant; PA, palmitate-BSA; sup, culture media.

We isolated thioglycollate-elicited peritoneal macrophages from Ldlr^−/−^ mice after 10 weeks of diet consumption. We treated macrophages with three different activators of the NLRP3 inflammasome: *a*) ATP, which activates P2X7 receptors and lowers intracellular K^+^ levels (30–32); *b*) oxidized LDL, which leads to cholesterol crystal formation and lysosomal disruption (6); and *c*) palmitate, which activates the inflammasome via an AMP-activated protein kinase-autophagy pathway (7). Compared with PO, macrophages from FO-fed and EO-fed, but not BO-fed, mice secreted significantly less IL-1β after stimulation with ATP (**Fig. 1A**, left panel). When stimulated with ox-LDL, recently shown to activate the NLRP3 inflammasome via CD36 and TLR4/TLR6 (6), macrophages from FO-, EO-, and BO-fed mice all had significantly lower IL-1β secretion (Fig. 1A, middle panel). Moreover, FO, EO, and BO markedly inhibited palmitate-induced IL-1β secretion (Fig. 1A, right panel). No IL-1β was detected in the culture supernatant of macrophages without LPS stimulation (data not shown), suggesting that these elicited macrophages were not inflamed. Similar results were observed when we used thioglycollate-elicited peritoneal macrophages from diet-fed C57BL/6 mice (data not shown).

Activation of the NLRP3 inflammasome is regulated by two-step signals (2, 33, 34). The first “priming” signal, such as LPS, enhances the transcription of inflammasome components such as pro-IL-1β and NLRP3 via activation of transcription factor NF-κB. The second “activation” signal promotes the assembly of inflammasome components and cleavage of caspase-1. To determine whether decreased IL-1β secretion results from blocking LPS-mediated priming and/or enhancing caspase-1 cleavage, we first conducted real-time PCR analysis to measure mRNA levels of IL-1β, IL-18, and NLRP3. Without LPS simulation, relative to PO, FO significantly reduced expression of macrophage IL-1β, IL-18, and IL-6 (a noninflammasomal cytokine) (Fig. 1B). BO also lowered IL-1β and IL-6 mRNA expression. As was expected, LPS priming (200 ng/ml LPS, 2 h) significantly upregulated expression of IL-1β, NLRP3, IL-18, and IL-6 via activation of NF-κb among all groups. Macrophages from mice fed dietary PUFAs (FO, EO, and BO) had decreased mRNA expression of IL-1β, IL-18, and IL-6. In contrast, expression of NLRP3 mRNA was significantly increased in EO-fed mouse macrophages after a 2-h LPS priming. Next, we analyzed caspase-1 cleavage using Western blotting. There was a marked decrease in caspase-1 cleavage in dietary PUFA-fed (FO, EO, and BO) versus PO-fed mouse macrophages in response to ATP (Fig. 1C) or palmitate stimulation (Fig. 1D). In summary, decreased IL-1β secretion from dietary PUFA-fed mouse macrophages results from attenuated IL-1β (but not NLRP3) mRNA expression and caspase-1 cleavage.

### Dietary PUFAs reduced hepatic inflammasome activation

**Fig. 2.**
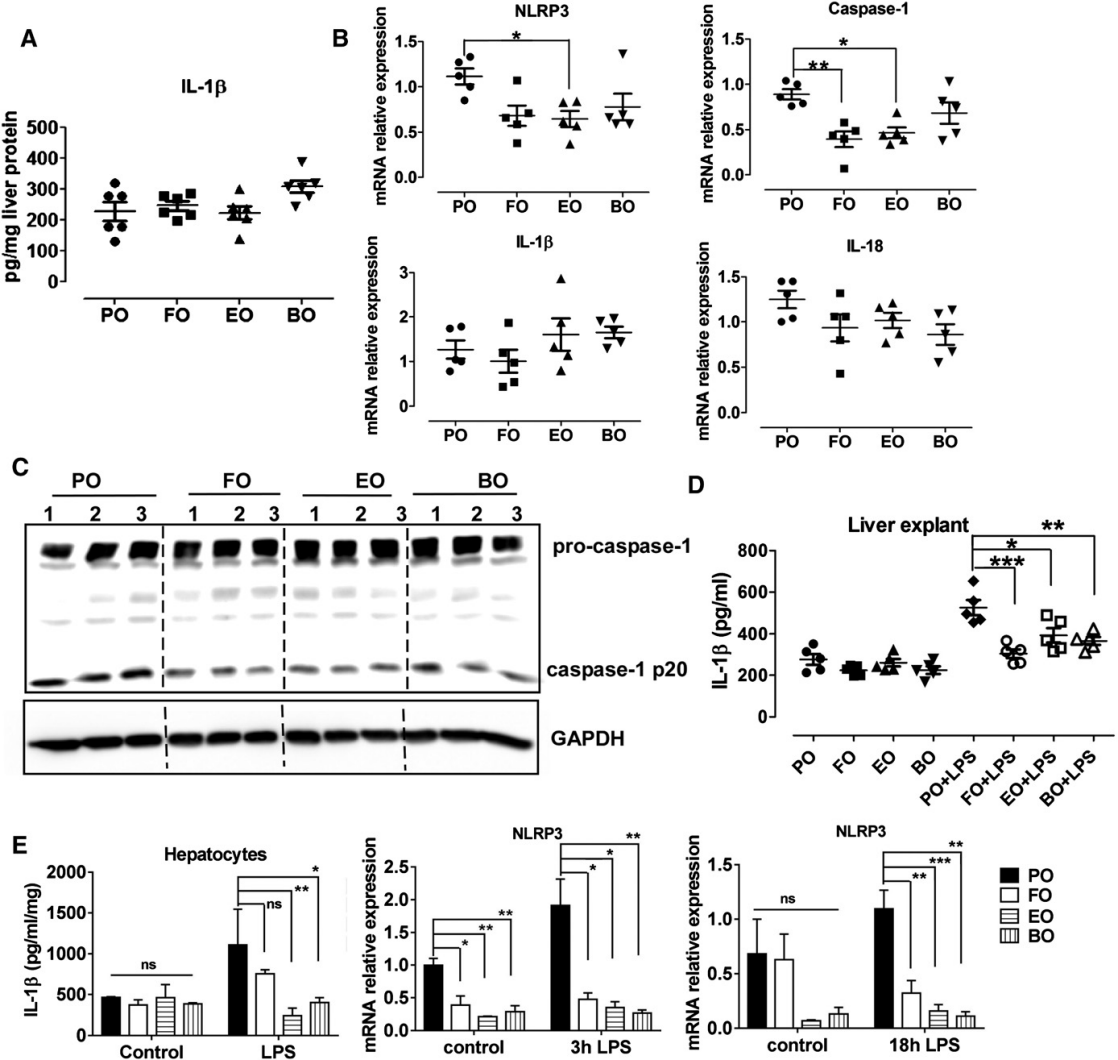
Dietary PUFAs reduced liver caspase-1 cleavage and IL-β secretion. A: IL-β concentrations in liver homogenates from female Ldlr^−/−^ mice fed atherogenic diets for 16 weeks. B: mRNA expression of NLRP3 inflammasome components in livers from female Ldlr^−/−^ mice fed atherogenic diets for 16 weeks was measured by real-time PCR. C: Immunoblotting analysis of caspase-1 cleavage in livers from female Ldlr^−/−^ mice fed diet for 16 weeks (n = 3 mice per diet group). D: Livers were isolated from female Ldlr^−/−^ mice fed diets for 10 weeks and cultured with or without 100 ng/LPS for 24 h. Culture supernatants were analyzed by ELISA for IL-1β. E: Hepatocytes were isolated from female Ldlr^−/−^ mice fed diets for 10 weeks and cultured in the presence or absence of 100 ng/LPS for 3 or 18 h. Culture supernatants were analyzed by ELISA for IL-1β, and cell lysates were analyzed by real-time PCR for NLRP3 mRNA expression. *P < 0.05; **P < 0.01; ***P < 0.001.

Inflammasome deficiency protects against high fat diet-induced hepatosteatosis, inflammation, and early fibrogenesis (35, 36). We previously reported that FO, EO, and BO lowered hepatosteatosis and inflammation compared with PO (23). We hypothesized that dietary PUFA supplementation attenuates hepatic steatosis by inhibiting atherogenic diet-induced activation of the inflammasome in vivo. To test this hypothesis, we first measured IL-1β concentrations in the liver homogenate from diet-fed Ldlr^−/−^ mice. IL-1β levels were indistinguishable among groups (**Fig. 2A**). However, FO, EO, or BO lowered mRNA expression of liver NLRP3 and capase-1 (statistically significant or a trend toward decrease) but not IL-1β or IL-18 (Fig. 2B). Immuno­blotting analysis of caspase-1 cleavage in liver from mice fed diet for 16 weeks (n = 3 mice per diet group) showed that the caspase-1 processing (i.e., cleavage) was significantly attenuated in livers from FO-, EO-, and BO-fed mice (compared with PO), detected as less caspase-1-p20 subunit expression (Fig. 2C). Next, we isolated livers from mice fed diets for 10 weeks and cultured in the presence or absence of 100 ng/LPS for 24 h. Consistent with macrophage results, livers from FO-, EO-, or BO-fed mice showed less IL-1β production in response to LPS (Fig. 2D). In addition to liver explants, primary hepatocytes isolated from dietary PUFAs (FO, EO, or BO) versus PO-fed mice also showed less IL-1β secretion and less NLRP3 mRNA expression in response to LPS (Fig. 2E). Taken together, compared with PO, dietary supplementation of PUFAs (FO, EO, and BO) significantly attenuated hepatic inflammasome activation, which may partially account for the reduced hepatic inflammation/steatosis observed in those mice.

### Dietary PUFAs reduced blood Ly6C^low^ monocyte caspase-1 activation

**Fig. 3.**
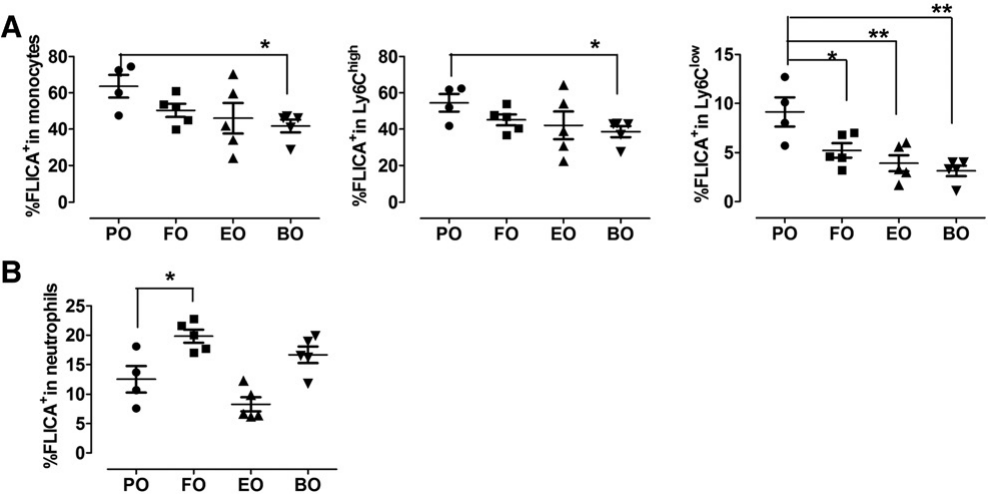
Caspase-1 cleavage in blood monocytes. Blood cells from Ldlr^−/−^ mice fed diets for 12 weeks were stained with PE-CD115, APC-Cy7-Gr1, and FAM-YVAD-FMK probe (FLICA). Percentage of FLICA^+^ cells in blood monocytes (CD115^+^ Ly6G^−^), Ly6C^low^ (CD115^+^Gr1^low^) and Ly6C^high^ (CD115^+^Gr1^high^) monocytes (A) and in neutrophils (CD115^−^ Ly6G^+^) (B) were analyzed by flow cytometry. Each symbol represents an individual mouse. *P < 0.05; **P < 0.01.

Palmitate induces and DHA inhibits inflammasome activation in human blood monocytes (37). To determine whether supplementation of FO, EO, or BO lowers blood monocyte activation in vivo, we used flow cytometry to measure active caspase-1 using FLICA dye in Ly6C^high^ and Ly6C^low^ blood monocytes (identified as CD115^+^Ly6G^−^) in 12-week diet-fed Ldlr^−/−^ mice. We observed relatively less staining of active caspase-1 (FLICA^+^) in blood monocytes, especially in Ly6C^low^ monocytes (**Fig. 3A**) but not in neutrophils (CD115^+^Ly6G^+^) (Fig. 3B) from dietary PUFA- versus PO-fed mice, indicating that dietary PUFAs specifically reduced blood Ly6C^low^ monocyte caspase-1 cleavage.

### Dietary PUFAs lowered LPS-induced IL-1β secretion in vivo

**Fig. 4.**
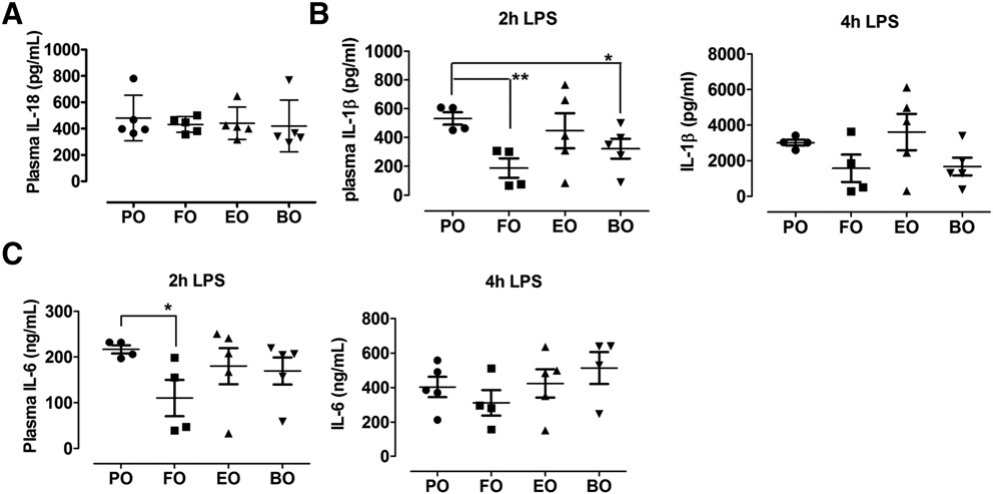
IL-18 or IL-1β concentrations in plasma. A: Plasma IL-18 concentrations in Ldlr^−/−^ mice fed diet for 16 weeks. B, C: Plasma IL-1β and IL-6 concentrations in Ldlr^−/−^ mice fed diet for 12 weeks and injected intraperitoneally with LPS (3 mg/kg). Plasma was isolated at 2 and 4 h postinjection. Each symbol represents an individual mouse. *P < 0.05; **P < 0.01.

Because we observed less inflammasome activation in peritoneal macrophages, blood monocytes, and hepatocytes from PUFA-fed mice, we next explored whether FO, EO, and BO supplementation can reduce LPS-induced proinflammatory cytokine production in vivo. We challenged diet-fed Ldlr^−/−^ mice a sublethal dose of LPS (3 mg/kg body weight) for 2 or 4 h and then measured plasma IL-1β concentrations. Prior to LPS injection, mouse plasma IL-18 concentrations were similar among groups (**Fig. 4A**) , and plasma IL-1β was undetectable. After 2 h of LPS challenge, FO-fed and BO-fed mice, but not EO-fed mice, had significantly less plasma IL-1β relative to PO. Similar trends were observed 4 h post-LPS injection (Fig. 4B). Only FO significantly lowered plasma IL-6 concentrations after 2 h of LPS injection (Fig. 4C).

### Dietary PUFAs enhanced autophagy activation

**Fig. 5.**
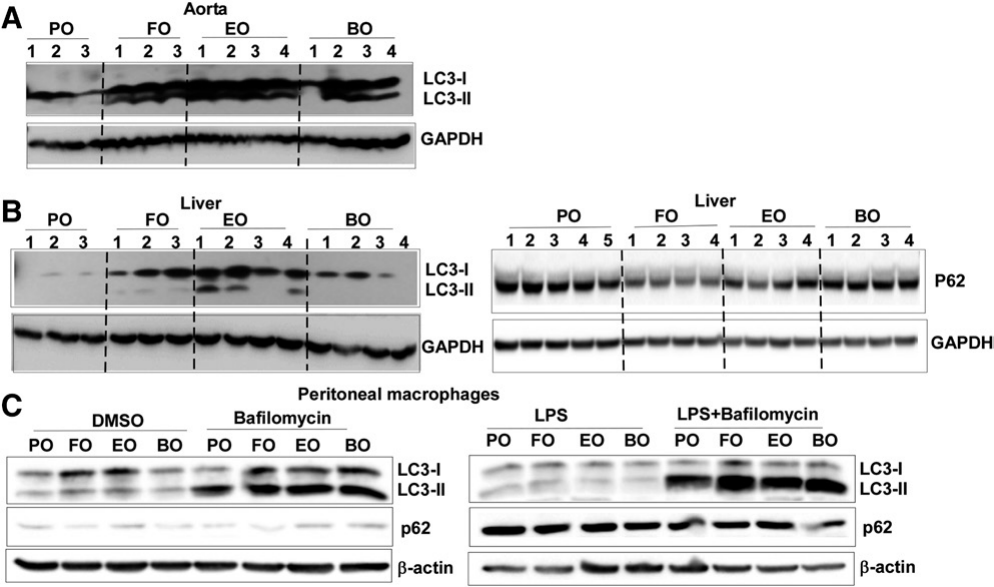
Dietary PUFAs enhanced autophagy. A, B: Immunoblotting analysis of LC3 and P62 expression in whole mouse aortas and livers from Ldlr^−/−^ mice fed diets for 16 weeks. C: Immunoblotting analysis of LC3 and P62 expression in thioglycollate-elicited peritoneal macrophages. Macrophages were isolated from C57BL/6 mice fed diets for 12 weeks, treated with or without 100 ng/ml LPS for 18 h with or without 50 nM bafilomycin A1.

DHA enhances macrophage autophagy in vitro (38). To examine whether dietary PUFA supplementation activates autophagy in vivo, we first analyzed LC3-II and p62/SQSTM1 expression by immunoblotting in livers and whole aortas from Ldlr^−/−^ mice fed atherogenic diets for 16 weeks. Relative to PO, dietary PUFAs (FO, EO, and BO) significantly increased expression of total LC3 and LC3-II in aortas (**Fig. 5A**) at 16 weeks of diet feeding. LC3-II increased slightly in livers from dietary PUFA-fed mice, but FO- and EO-fed mice showed significantly less liver P62 expression (Fig. 5B), suggesting enhanced autophagy activation. Next, we assessed autophagic flux in macrophages. Briefly, thioglycollate-elicited peritoneal macrophages were isolated and stimulated with or without 100 ng/ml LPS for 18 h in the presence or absence of bafilomycin, an inhibitor of autophagosome-lysosome fusion. As is shown in Fig. 5C, compared with PO, dietary PUFAs (FO, EO, or BO) increased macrophage LC3-II expression, suggesting increased autophagic flux. P62 expression was increased after LPS stimulation, as has been previously reported (39), to a consistent degree among groups. Taken together, our results indicate that dietary PUFAs enhanced autophagy activation in tissues and macrophages.

### Macrophage autophagy deficiency abolished PUFA-induced inflammasome inhibition

**Fig. 6.**
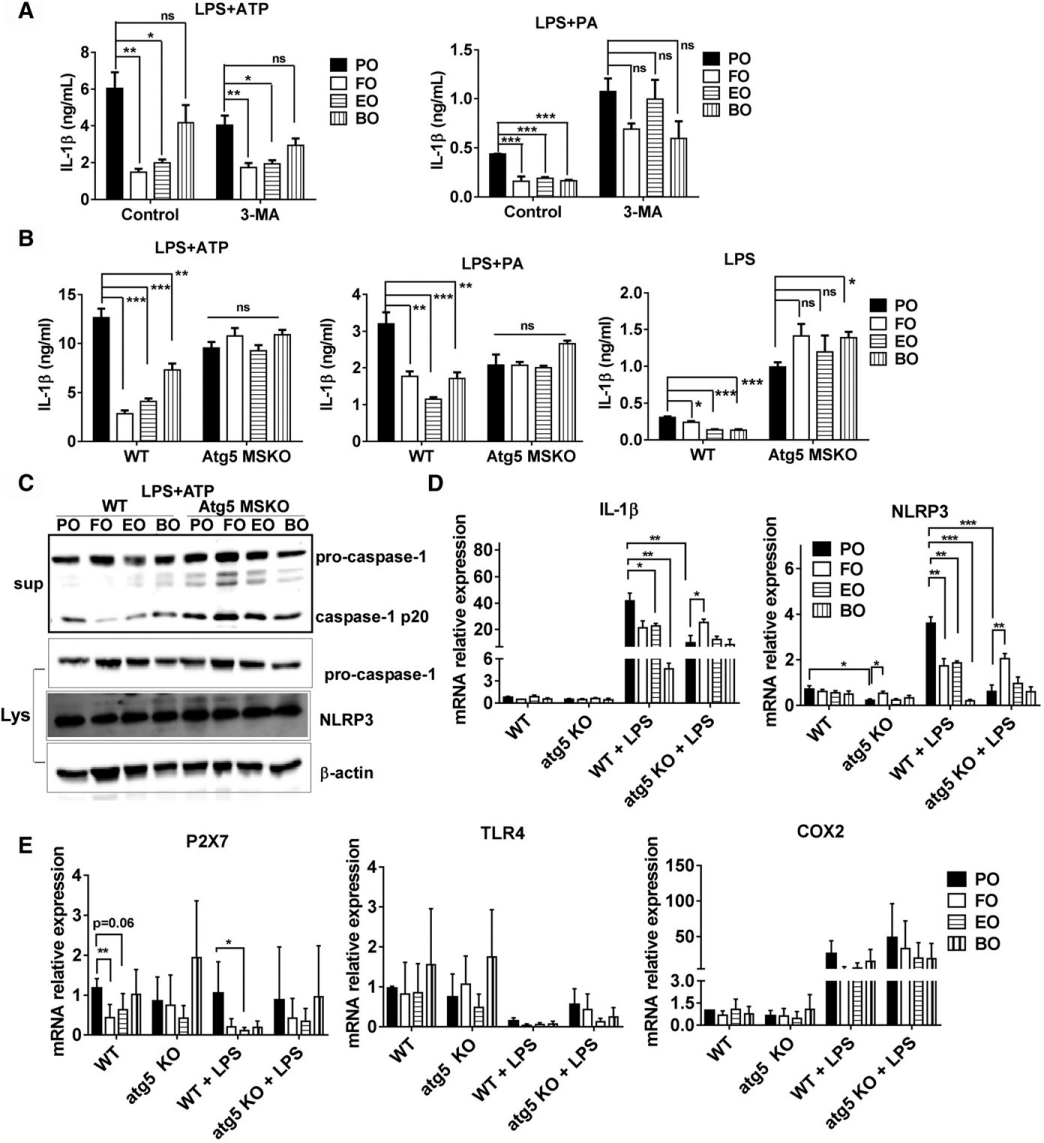
Macrophage autophagy deficiency abolishes PUFA-induced inflammasome inhibition. A: Thioglycollate-elicited peritoneal macrophages were isolated from C57BL/6 mice fed diets for 12 weeks, primed with 200 ng/ml LPS for 2 h, and treated with 5 mM ATP for 1 h or 200 μM palmitate-BSA for 24 h with or without 3-methyladenine (3-MA; 5 mM). IL-1β secretion in culture media was assayed using ELISA. B, C: Thioglycollate-elicited peritoneal macrophages were isolated from WT and macrophage-specific atg5 KO (atg5 MSKO) mice fed diet for 12 weeks, primed with 200 ng/ml LPS for 2 h, and treated with 5 mM ATP for 1 h or 200 μM palmitate-BSA for 24 h. IL-1β secretion in culture media was assayed using ELISA (B) and protein expression in LPS plus ATP-treated macrophages was assayed by immunoblotting (C). D, E: Irradiated Ldlr^−/−^ mice were transplanted with WT and atg5 MSKO bone marrow and fed diets for 16 weeks. Resident peritoneal macrophages were isolated and treated with or without LPS (100 ng/ml) for 3 h. Macrophage gene expression was analyzed by real-time PCR *P < 0.05; **P < 0.01; ***P < 0.001.

To determine the role of autophagy in PUFA-mediated inflammasome inactivation, we first measured NLRP3 inflammasome activation in the absence or presence of the autophagy inhibitor 3-methyladenine (3-MA) using peritoneal macrophages from 10-week diet-fed WT mice. Consistent with results described above, dietary PUFAs attenuated ATP- and palmitate-induced IL-1β production (**Fig. 6A**). However, 3-MA did not significantly abolish ATP-induced IL-1β secretion and only partially reduced palmitate-mediated IL-1β secretion (Fig. 6A).

Because most drugs have off-target effects, we next used macrophages from diet-fed WT and atg5 MSKO mice to examine inflammasome activation. As is shown in Fig. 6B, IL-1β levels in macrophages from dietary PUFA- and PO-fed atg5 MSKO mice were similar in response to ATP or palmitate, suggesting that auto­phagy is a key regulator in PUFA-mediated NLRP3 inflammasome inactivation. LPS priming significantly increased IL-1β production in atg5 MSKO than in WT macrophages; this effect was more profound in dietary PUFA-fed mouse macrophages (Fig. 6B, right panel).

To further explore the role of autophagy in PUFA-mediated inflammasome inactivation, we analyzed caspase-1 cleavage and NLRP3 protein expression using Western blotting. As is shown in Fig. 6C, dietary PUFAs (FO, EO, and BO) markedly decreased caspase-1 cleavage in WT macrophages in response to ATP. In contrast, in atg5 MSKO macrophages, PUFAs did not reduce caspase-1 cleavage, shown by relatively similar or slightly higher caspase-1 p20 secretion in PUFA than in PO macrophages. There was no significant difference in NLRP3 protein expression among groups. Next, we conducted real-time PCR analysis to measure mRNA levels of IL-1β, NLRP3, TLR4, and P2X7 (ATP receptor) in resident peritoneal macrophages from 16- week diet-fed irradiated Ldlr^−/−^ mice transplanted with bone marrow from WT or atg5 MSKO mice (Fig. 6D). Without LPS simulation, expression of IL-1β or NLRP3 did not differ among dietary PUFAs (FO, EO, and BO) versus PO-fed mouse WT macrophages. Unexpectedly, in the absence of LPS stimulation, there was a significant reduction of NLRP3 mRNA expression in PO-fed atg5 KO versus WT macrophages. Interestingly, FO versus PO significantly increased NLRP3 mRNA expression in atg5 KO macrophages without LPS stimulation. Upon LPS (100 ng/ml LPS, 3 h) stimulation, there was significantly upregulated mRNA expression of IL-1β and NLRP3 in macrophages among all groups. Macrophages from WT mice fed dietary PUFAs (FO, EO, and BO) had significantly decreased or showed a trend toward decease in mRNA expression of IL-1β (FO; *P* = 0.0579) and NLRP3. In contrast, atg5 KO macrophages from dietary PUFA- fed (FO, EO, and BO) mice showed no difference (EO and BO) or an increase (FO) in mRNA expression of IL-1β and NLRP3.

We also observed that relative to PO, FO significantly lowered P2X7 receptor expression in WT but not atg5 KO macrophages. EO marginally reduced P2X7 receptor expression, and BO had no effect (Fig. 6E, left panel). After LPS challenge, all three PUFAs reduced P2X7 expression in WT but not atg5 KO macrophages compared with PO (only reaching statistical significance in the EO group). TLR4 expression did not differ among the groups or between genotypes (Fig. 6E, middle panel). Additionally, no significant differences were observed in COX2 mRNA expression in macrophages among groups (Fig. 6E, right panel).

Taken together, our results suggest that autophagy plays a critical role in PUFA-induced inflammasome inhibition by regulating both the priming (decreasing IL-1β and NLRP3 mRNA) and secondary inflammasome stimulation (decreasing caspase-1 cleavage) steps. Moreover, n-3 (FO and EO) but not n-6 (BO) PUFAs downregulate macrophage ATP receptor P2X7 expression, which may explain the lower inflammasome inactivation in BO-fed mouse macrophages in response to ATP.

### Dietary PUFAs restored mitochondrial function

**Fig. 7.**
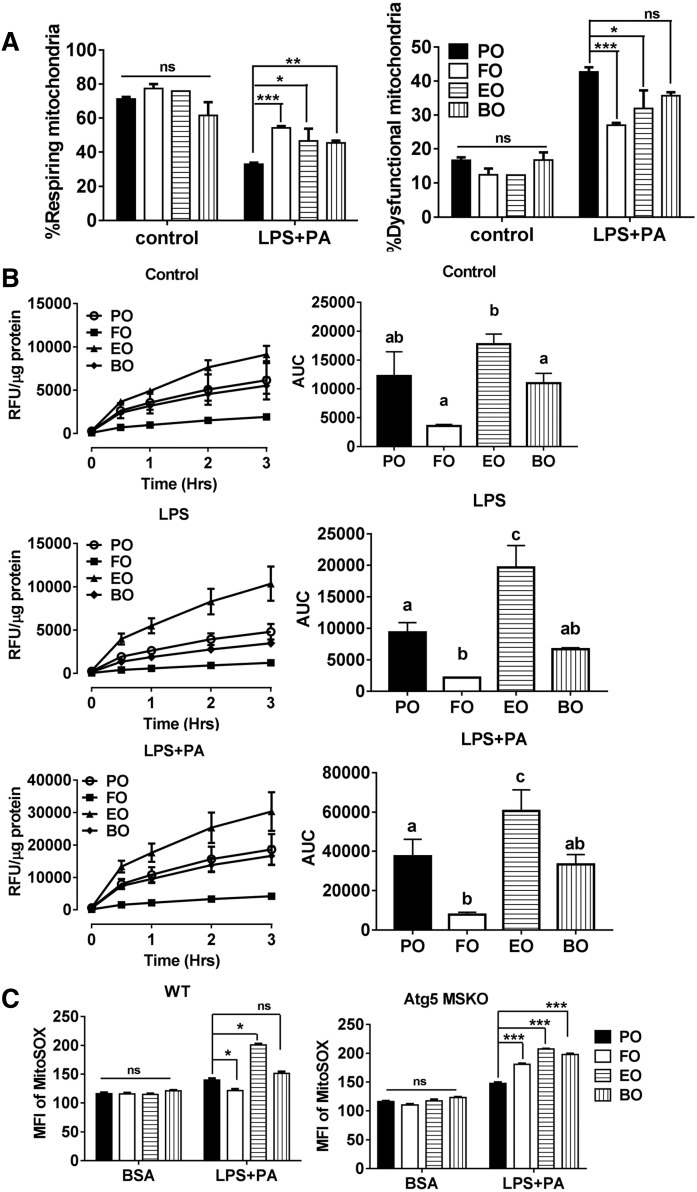
Mitochondrial function and production of ROS. A: Thioglycollate-elicited peritoneal macrophages from C57BL/6 mice fed diets for 12 weeks were primed with 200 ng/ml LPS for 2 h and treated with 200 μM palmitate-BSA for 24 h. Cells were stained with Mitotracker Green and Mitotracker Deep red for 15 min and analyzed by flow cytometry. B: Thioglycollate-elicited peritoneal macrophages from C57BL/6 mice fed diets for 12 weeks were primed with 200 ng/ml LPS for 2 h and treated with 200 μM palmitate-BSA in the presence of 50 μM DCFDA (5-(and 6)-chloromethyl-2’,7’-dichlorodihrofluorescein diacetate, acetyl ester) for 3 h. ROS production was measured using a microplate reader and expressed as area under the curve. C: Thioglycollate-elicited peritoneal macrophages from WT and macrophage-specific atg5 KO (atg5 MSKO) mice fed diets for 12 weeks, treated with or without LPS+PA as described in panel A. Cells were stained with MitoSOX for 30 min and then analyzed by flow cytometry. Groups with different letters are significantly different (*P* < 0.05). *P < 0.05; **P < 0.01; ***P < 0.001. AUC, area under the curve; MFI, mean fluorescent intensity; RFU, relative fluorescence units.

Autophagy is critical for clearance of dysfunctional mitochondria and is a negative regulator of the NLRP3 inflammasome (14, 15). Because we observed increased autophagic influx in dietary PUFA-fed mouse macrophages, we hypothesized that dietary PUFAs may improve mitochondrial function and thus reduce NLRP3 inflammasome activation. To test this hypothesis, we first stained the functional mitochondria with MitoTracker Deep Red, a fluorescent probe sensitive to the mitochondrial inner transmembrane potential. We counterstained total mitochondria with MitoTracker Green, a probe that stains mitochondrial membrane lipids independently of membrane potential. As is shown in **Fig. 7A**, , treatment with LPS plus palmitate markedly decreased the percentage of mitochondrial respiration (high positive stain for MitoTracker Deep Red) in all diet groups. Compared with PO, dietary PUFAs (FO, EO, and BO) significantly increased the percentage of respiring mitochondria and lowered the percentage of dysfunctional mitochondria, suggesting that dietary PUFAs improved mitochondrial function.

### FO, but not EO and BO, reduced macrophage production of ROS

Because ROS, especially mitochondrial ROS, are essential for inflammasome activation (4, 15, 40, 41), we next investigated whether increasing the proportion of respiring mitochondria lead to lower ROS production in PUFA-fed mouse macrophages. We measured intracellular ROS production using DCFDA and mitochondrial ROS production using MitoSOX (a mitochondrial superoxide indicator). LPS or LPS plus palmitate induced ROS production in all diet-fed mouse macrophages (Fig. 7B). However, only FO-fed (vs. PO fed) mouse macrophages had attenuated intracellular ROS. Unexpectedly, EO-fed mouse macrophages had significantly higher ROS (Fig. 7B). A similar trend was found in mitochondrial ROS production (Fig. 7C, left panel). Mitochondrial ROS production induced by LPS plus palmitate was significantly upregulated in macrophages from PUFA-fed atg5 MSKO mice, relative to PO (Fig. 7C, right panel). Together, these results suggest that despite increased autophagy and mitochondrial function, dietary n-3 and n-6 PUFAs (but not FO) do not lower mitochondrial ROS production in WT macrophages.

## DISCUSSION

We tested the hypothesis that dietary enrichment of botanically derived PUFAs beyond D6D, a rate-limiting enzyme in essential polyunsaturated FA biosynthesis (EO and BO), reduces liver steatosis and atherosclerosis via attenuation of macrophage inflammasome activation. To date, only one study has shown that gavage of DHA in high-fat diet-fed mice attenuated NLRP3 inflammasome activation and prevented mice from high-fat diet-induced insulin resistance at a whole-body level (42). However, it remains unknown whether consumption of dietary n-3 or n-6 PUFAs beyond D6D inhibits macrophage inflammasome activation in vivo. Here, we showed that dietary n-3 (FO and EO) and n-6 (BO) PUFAs supplementation suppressed both LPS priming and NLRP3 inflammasome activation (assessed as caspase-1 cleavage) in macrophages, blood monocytes, and hepatocytes. PUFAs repressed NLRP3 inflammasome activation via activation of autophagy. Furthermore, n-3 and n-6 PUFAs supplementation improved macrophage mitochondrial function but did not significantly reduce mitochondrial ROS production (except FO). Together, our data suggest that dietary n-3 and n-6 PUFAs suppress NLRP3 inflammasome activation via enhancing autophagy and mitochondrial function, independent of mitochondrial ROS production.

The macrophage NLRP3 inflammasome has been implicated in the pathogenesis of metabolic diseases, including type 2 diabetes and atherosclerosis. For example, Ldlr^−/−^ mice that received bone marrow from NLRP3^−/−^, ASC^−/−^, or IL-1α/β^−/−^ mice developed less atherosclerosis after 8 weeks of a high-cholesterol diet, relative to control mice, suggesting that activation of the NLRP3 inflammasome in bone marrow-derived cells contributes to diet-induced atherosclerosis (4). Increasing evidence suggests that n-3 PUFAs inactivate macrophage NLRP3 inflammasome activation (26, 42–44). In term of mechanisms, Yan et al. (42) showed that n-3 FAs (including DHA and EPA) signal through the G protein-coupled receptor 120 (GPR120) and GPR40 to inhibit macrophage NLRP3 inflammasome activation. They found that n-6 FAs (including linoleic acid, DGLA, and AA) and n-9 FAs (including oleic acid) failed to block IL-1β secretion induced by nigericin. Hence, they concluded that only certain n-3 FAs inhibit macrophage NLRP3 inflammasome activation induced by nigericin (42). In addition, β-arrestin 2 acted downstream of GPR120 and GPR40 to inhibit inflammasome activation via directly binding with NLRP3. Deletion of β-arrestin 2 only partially inhibited DHA activity, suggesting that another mechanism independent of GPR120/40 and β-arrestin 2 might be involved in inhibiting activation of the NLRP3 inflammasome.

Our previous studies demonstrated that botanical oils enriched in 18:4 n-3 (EO, botanical sources of n-3 PUFAs, endogenously rapidly converted to EPA) and 18:3 n-6 (BO, botanical sources of n-6 PUFAs, endogenously rapidly converted to AA) are as atheroprotective as FO (animal sources of n-3 PUFAs, enriched in EPA, and DHA) compared with saturated/monounsaturated fat enriched PO in Ldlr^−/−^ mice (22–24, 45, 46). In the current study, we used peritoneal macrophages isolated from PO-, FO-, EO-, and BO-fed mice and examined NLRP3 inflammasome activation in response to ATP, oxLDL, or palmitate. Dietary enrichment of 18-carbon FAs beyond D6D resulted in membrane enrichment in their respective 20- or 22-carbon chain counterparts in macrophages (Table 2). Compared with n-6 PUFAs (BO), n-3 PUFAs (FO and EO) were more potent in inhibiting ATP-mediated NLRP3 inflammasome (Figs. 1, 6). Both n-3 PUFAs (FO and EO) and n-6 PUFAs (BO) significantly attenuated ox-LDL- or palmitate-induced activation of the macrophage NLRP3 inflammasome, relative to PO (Fig. 1). Decreased IL-1β secretion from dietary PUFA-fed mouse macrophages resulted from attenuated IL-1β and NLRP3 mRNA expression and caspase-1 cleavage. Furthermore, ly6C^low^ monocytes from dietary PUFA-fed mice had lower caspase-1 activity relative to PO-fed mice. N-6 PUFAs (BO) seemed to have a more profound inhibitory effect on caspase-1 cleavage in blood monocyte (Fig. 3). Despite differential effects of PUFAs, in general, consumption of dietary n-3 or n-6 PUFAs reduces monocyte/macrophage NLRP3 inflammasome activation in vivo.

Increasing evidence suggests that inflammasome activation enhances diet-induced nonalcoholic steatohepatitis (NASH). For example, mice deficient in caspase-1 exhibited less high fat diet-induced hepatic steatosis, inflammation, and early fibrogenesis (35). Macrophage-specific caspase-1/11 deficiency protects against cholesterol crystal formation and hepatic inflammation via enhanced cholesterol efflux and autophagy (36). Conversely, activation of the NLRP3 inflammasome enhances liver inflammation and fibrosis (47). TLR2 and palmitic acid cooperatively activate the inflammasome in Kupffer cells, contributing to development of NASH (48). IL-1β signaling is required for development of alcohol-induced liver steatosis, inflammation, and injury, attributed to inflammasome activation in bone marrow-derived Kupffer cells (49). We previously reported that dietary FO, EO, and BO versus PO supplementation lowered hepatic steatosis and inflammatory gene expression (23), but the mechanisms were unclear. In the current study, we found that livers from mice fed dietary PUFAs (FO, EO, or BO) versus PO exhibited less caspase-1 cleavage (Fig. 2). Additionally, less IL-1β secretion was observed in liver explants and hepatocytes isolated from mice fed dietary PUFAs (FO, EO, or BO) versus PO-fed mice, as well as less NLRP3 mRNA expression in response to LPS. We did not use a Percoll density gradient to remove Kupffer cells, so we cannot rule out the presence of other cells (e.g., Kupffer cells) in our hepatocyte cultures. Nevertheless, our results suggest that dietary PUFAs attenuated hepatic NLRP3 inflammasome activation (Fig. 2), which in part explains the reduced hepatic steatosis in mice fed dietary PUFAs.

Autophagy, upregulated by pathogens and/or nutrient deprivation, is a major innate defense pathway in immunity and inflammation (50). Using a higher concentration of DHA (50 or 100 μM) in an in vitro experiment, Williams-Bey et al. (38) showed that DHA suppressed NLRP3 inflammasome by inhibiting NF-κb activation and enhancing autophagy in macrophages. In the current study, botanical sources of PUFAs enrichment beyond D6D (EO and BO) enhanced macrophage and tissue (aorta and liver) autophagy (Fig. 5). Furthermore, genetic inhibition of macrophage autophagy abolished dietary PUFA-mediated NLRP3 inflammasome inactivation (Fig. 6), suggesting that consumption of dietary n-3 or n-6 PUFAs inhibits the NLRP3 inflammasome by enhancing autophagy activation. To further explore the role of autophagy in PUFA-mediated inflammasome inactivation, we analyzed caspase-1 cleavage, NLRP3 mRNA/protein, TLR4, or P2X7 mRNA expression in PUFA-fed versus PO-fed WT and atg5 KO mouse macrophages. PUFAs did not reduce caspase-1 cleavage or lower IL-1β or NLRP3 mRNA expression, suggest that autophagy regulates PUFA-induced inflammasome inhibition by acting on both the priming (decreasing IL-1β and NLRP3 mRNA) and secondary inflammasome stimulation (decreasing caspase-1 cleavage) steps. Additionally, n-3 PUFAs (FO and EO) reduced P2rx7 receptor in WT but not in atg5 KO macrophages, which partially explains the more potent inhibitory effects of FO and EO on ATP-induced inflammasome activation in compared with BO (Fig. 6).

Increasing evidence suggests that mitochondria are at the center of NLRP3 inflammasome activation (51). First, mitochondria are the major sources of cellular ROS. Inhibition of mitochondrial complex I by rotenone or complex III by antimycin A induces robust mitochondrial ROS production, which drives NLRP3 inflammasome activation. Also, ATP, monosodium urate crystal, silica, and asbestos particles induce ROS production and activate NLRP3 inflammasomes (40, 41, 52). Conversely, treatment of macrophages with ROS inhibitors inhibits NLRP3 inflammasome activation (52). Second, mitochondrial destabilization induces cardiolipin, an inner mitochondrial membrane phospholipid, to move to the outer mitochondrial membrane (53). Cardiolipin directly binds to the leucine-rich repeats of NLRP3 and activates the NLRP3 inflammasome (54). Mitochondrial DNA in the cytoplasm activates the NLRP3 inflammasome (14, 55). Mitochondrial ROS is required for release of mitochondrial DNA and activation of the NLRP3 inflammasome (14). However, ROS activation is not an absolute requirement for activation of all NLRP3 inflammasomes. In particular, stimulation of macrophages with linezolid (from the oxazolidinone class of antibiotics) or infection of macrophages with influenza and encephalomyocarditis viruses does not require ROS for activation of the NLRP3 inflammasome (54, 56).

In the current study, we observed that although all dietary PUFAs improved macrophage mitochondrial function compared with PO, only FO-fed mouse macrophages had attenuated cellular/mitochondrial ROS production (Fig. 7). Unexpected, EO-fed mouse macrophages had significantly higher cellular/mitochondrial ROS production, relative to others, suggesting that the attenuated NLRP3 inflammasome activation in macrophages is independent of mitochondrial ROS. The underlying mechanisms that explain these differential effects need to be further explored. It is also unknown whether dietary PUFAs could differentially downregulate cardiolipilin externalization to the outer membrane of the mitochondria and thus attenuate activation of the NLRP3 inflammasome in macrophages.

Using a targeted lipidomics approach, Norris and Dennis (57) showed that AA, EPA, and DHA supplementation altered membrane phospholipid PUFA composition and subsequent PUFA release and eicosanoid production in Raw264.7 macrophages after TLR4 and purinergic receptor activation. They also showed that both n-3- and n-6-supplemented 22-carbon FAs had differential inhibitory properties on cyclooxygenase metabolism and that EPA-derived DPA is likely a major source of inhibition (58, 59). In our study, FO supplementation increased EPA, DHA, and DPA enrichment; EO increased EPA and DPA but not DHA enrichment; and BO increased DGLA and AA enrichment in liver and macrophages, respectively (Tables 2, 3). DPA, which is a potent cyclooxygenase inhibitor (58) and a precursor of resolvin-like anti-inflammatory molecules (59), was the predominant n-3 PUFA increased in FO- and EO-fed mouse macrophages (Table 2), which agreed with previous findings that EPA supplementation predominantly enhances DPA enrichment in macrophages (57). Additionally, LPS priming and ATP stimulation activate a set of phospholipases with different specificities to be coupled with subsequent eicosanoid formation. We did not measure eicosanoid production in dietary-fed mouse macro­phages. Although macrophage cyclooxygenase-2 expression did not differ among diets (Fig. 6), we cannot rule out the possibility that formation of various eicosanoids and other lipid mediators in macrophages account for differential effects of n-3 (FO and EO) and n-6 PUFAs (BO) on inflammasome activation and/or mitochondrial function.

In summary, our studies add novel data regarding protective mechanisms of dietary PUFA supplementation in cardiovascular disease, including activation of macrophage autophagy, improvement of mitochondrial function, and attenuation of the NLRP3 inflammasome activation. These results may inform future dietary recommendations to reduce cardiovascular disease and promote public health.
